# Supplementary material for: X under Musk’s leadership: Substantial hate and no reduction in inauthentic activity
Source: PLoS One. 2025 Feb 12;20(2):e0313293. doi: 10.1371/journal.pone.0313293 (PMC11819532; doi:10.1371/journal.pone.0313293)
Supplement: S1 File — We show example hate speech and coordinated account posts, as well as robustness checks of our results. (PDF) [file pone.0313293.s001.pdf]

Supporting Information for: X Under Musk's Leadership: Substantial Hate and No Reduction in Inauthentic Activity

Daniel Hickey[1], Daniel M.T. Fessler[2] Kristina Lerman[3] Keith Burghardt[3*],

1 School of Information, University of California, Berkeley, Berkeley, California, USA
2 Department of Anthropology and UCLA Bedari Kindness Institute, University of California, Los Angeles, Los Angeles, California, USA
3 USC Information Sciences Institute, Marina del Rey, California, USA

* keithab@isi.edu

Table S1. Example posts for each type of hate speech.

Homophobic	i hate this f****t
	@Anonymous Theres a f****t where I work, he is just disgust degenert. Every day i see him i became more homophobic https://t.co/xxxx
Racist	like wait until those n****r g*ks turn 100 THEY WILL CQLL THAT JAP THE N****R TIMES X
	@Anonymous Can't ban from Reddit for saying 'n****r' here. Also indians smell bad and don't have toilets. Burrnnnnned!
Transphobic	@Anonymous This is depressing but how stupid do your parents have to be to allow you to go down this T****y rabbit hole. You can't change your gender you can just become a freak that looks terrible in either catagory
	I dont want to risk my privacy bc of some stupid t****y https://t.co/xxxx

When applying robustness checks, we find that hate speech when including the r-slur is 1.90 times greater post-Musk (excluding the big spike) (Mann-Whitney U test p-value < 0.001). Hate speech likes is 2.23 times greater after Musk (Mann-Whitney U test p-value < 0.001), and hate speech reposts are 2.79 times greater post-Musk, (Mann-Whitney U test p-value $= 0.002$). In addition, there are insigificant results, such as 1.11 times as many likes per post (Mann-Whitney U test p-value $= 0.9$), and 1.43 times as many reposts per post (Mann-Whitney U test p-value $= 0.06$).

Via the Identity attack model, we find hate posts are 1.53 times more common (Mann-Whitney U test p-value < 0.001), hate post likes are 4.58 times greater (Mann-Whitney U test p-value $= 0.04$), and hate post reposts are 2.42 times greater

Table S2. Example posts for each coordination network.

Hashtag co-occurrence	@Anonymous @Anonymous Do you know one thing? Hot people listen to #YetToCome by #BTS from the album #BTS_Proof. Honey, @Anonymous is the blueprint.
Co-repost similarity	@Anonymous @Anonymous DO YOUR THANG ARMY Do you know one thing? Hot people listen to #YetToCome by #BTS from the album #BTS_Proof. Honey, @Anonymous is the blueprint. Nove<br>Prodigies are working overtime to #SaveProdigalSon We want our people back! We need our crime drama back!!! @Anonymous please help us with Prodigal Son Seasons 3-10! #BadDadKids I can tell you they are looking at Malcolm by the expressions on their faces! https://xxxx
Activity similarity	Great representation, relevant real world issues & complex flawed characters - #ProdigalSon is a cut above the average procedural #MurderNonsense. With so many stories still left to tell, worldwide fans are eager to to see someone #SaveProdigalSon! 65<br>Top 5 noticeable movers: #REP #DODO #UNFI #AKRO #BEL $REP $11.82 (28.48%) $DODO $0.19 (38.69%) $UNFI $3.109 (57.50%) $AKRO $0.00635 (89.55%) $BEL $1.104 (215.43%) Do you #hodl any of these coins?
	Full Financial Robot for #Forex #Trading #Signal on #Forex and #indices #cfd, live with #mt4 since 2010, for #metatrader4 with any broker #metatrader, go to https://yyyy https://xxxx

(Mann-Whitney U test p-value = 0.2). In addition, there are insigificant results, namely reposts per post are 1.83 times greater (Mann-Whitney U test p-value = 0.8), and likes per post are 3.4 times greater (Mann-Whitney U test p-value = 0.7).

For data in which we do not downsample posts before December 1, 2022, there are

1.08 times as many posts per week (Mann-Whitney U test p-value < 0.001), which is the same as in the main text, 13% more likes per week (Mann-Whitney U test p-value $= 0.01$), 21% fewer reposts per week (Mann-Whitney U test p-value $= 0.01$), 4% more likes per post (Mann-Whitney U test p-value< 0.001), and 23% fewer reposts per post (Mann-Whitney U test p-value < 0.001).
