# Supplementary figures and images for: X under Musk’s leadership: Substantial hate and no reduction in inauthentic activity
Source: PLoS One. 2025 Feb 12;20(2):e0313293. doi: 10.1371/journal.pone.0313293 (PMC11819532; doi:10.1371/journal.pone.0313293)

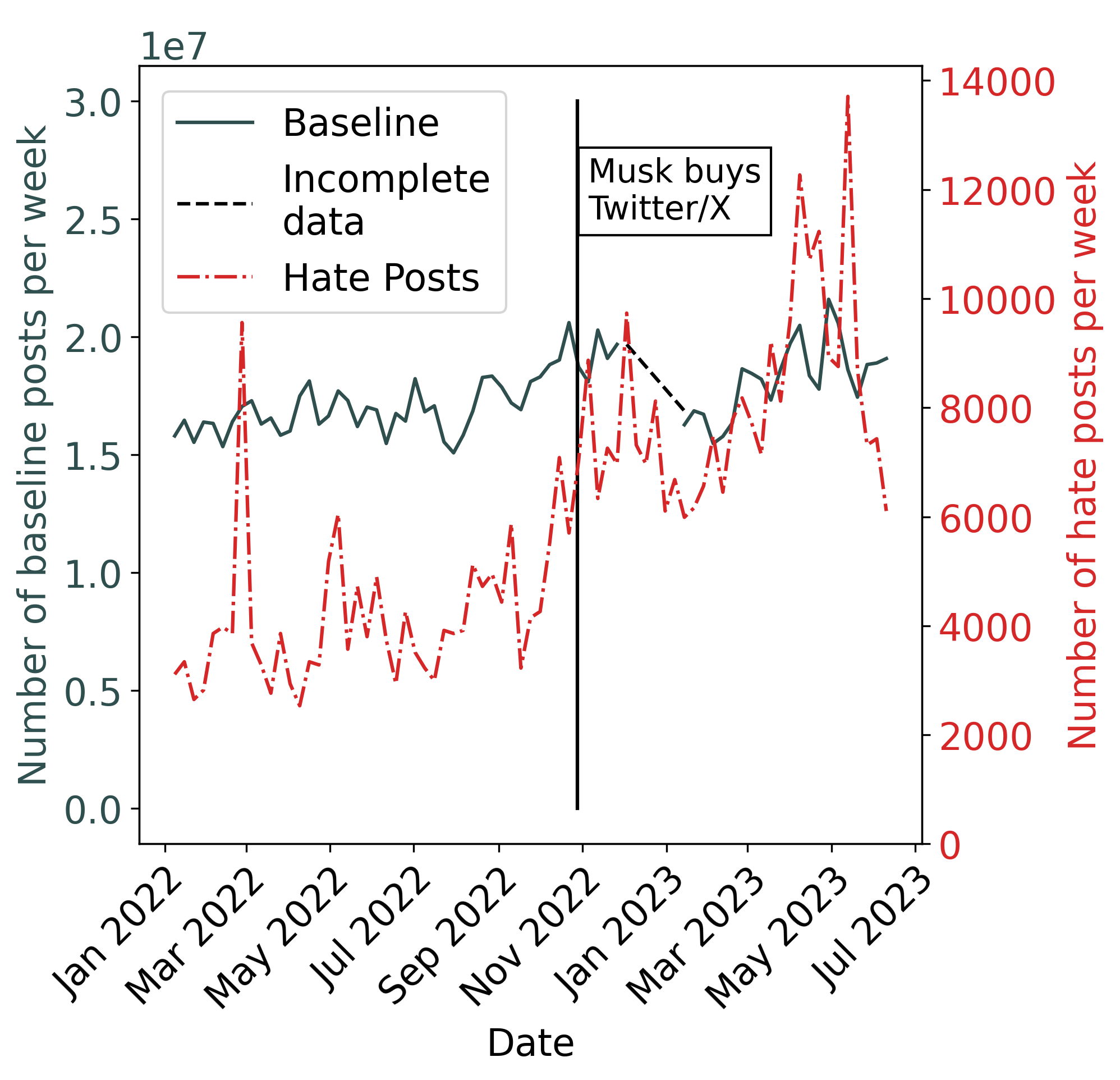

Supplement: S1 Fig — (PNG) [file pone.0313293.s002.png]

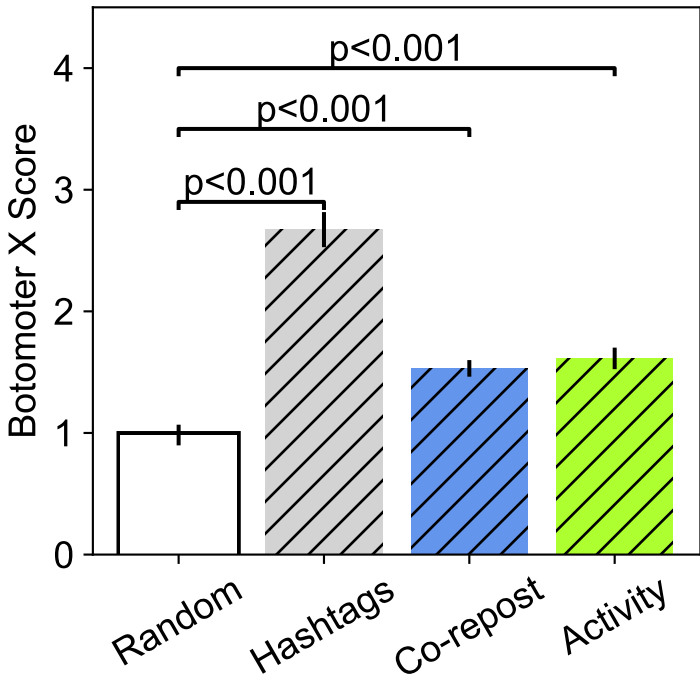

Supplement: S2 Fig — Bot scores are significantly higher in coordinated accounts (above brackets are Mann-Whitney U test p-values) when we measure 100 random accounts, 100 accounts coordinated by hashtag sequences, 100 accounts coordinated by co-repost behavior, and 100 accounts coordinated by activity. All accounts are sampled at random regardless of their activity. (PDF) [file pone.0313293.s003.pdf]

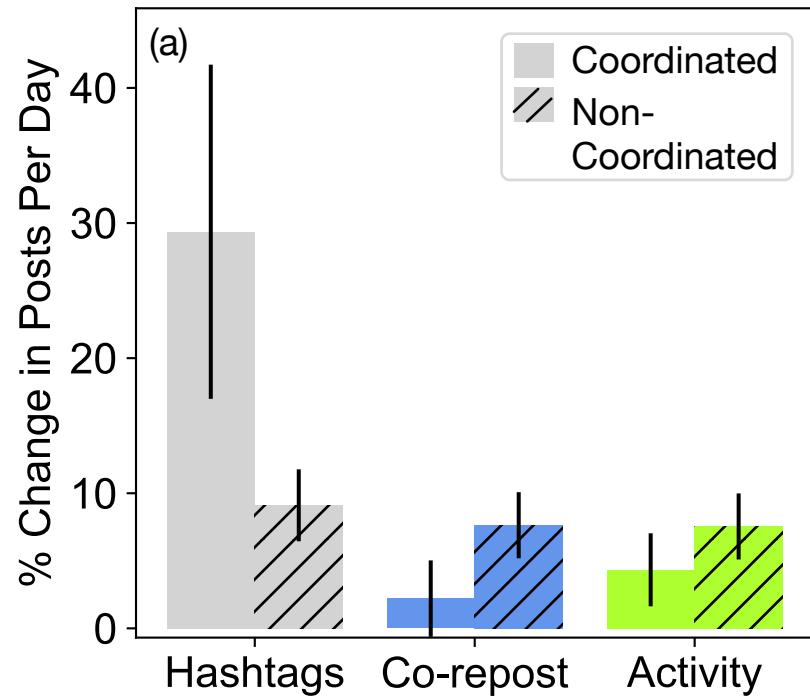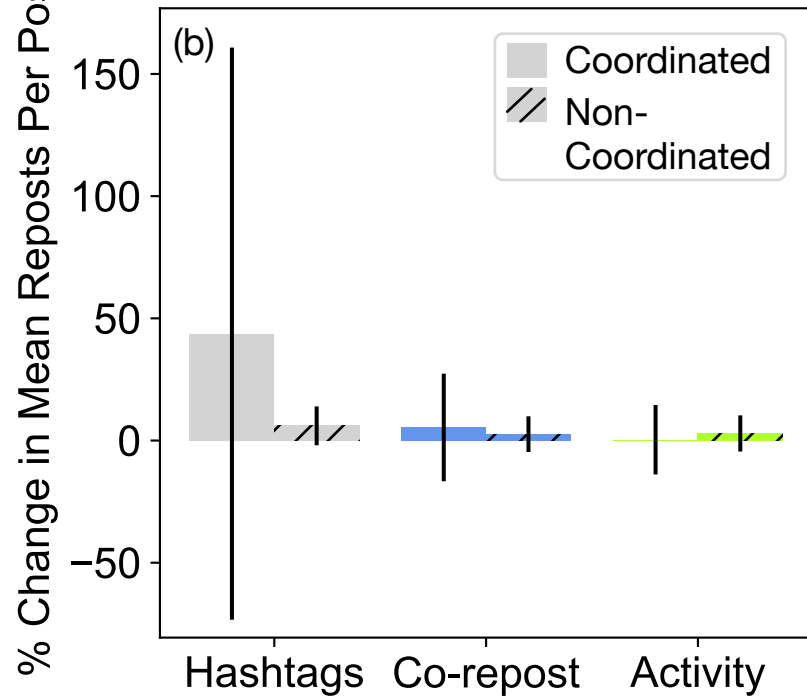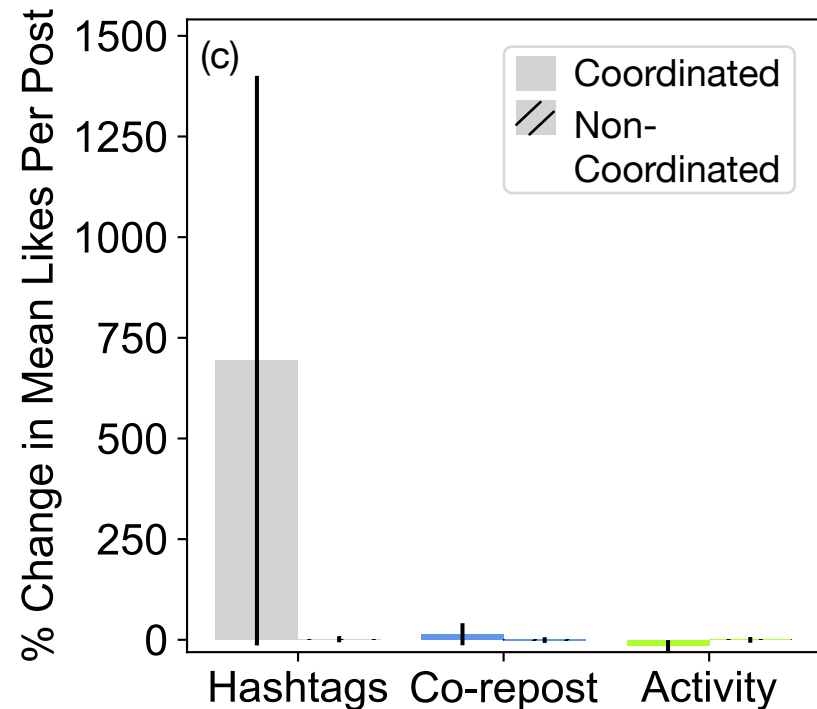

Supplement: S3 Fig — We use 3 different coordination metrics: hashtag sequence [32], co-repost [32, 33], and activity [32, 33]. Accounts are coordinated if they share a sequence of at least 5 hashtags in a post or have a co-retweet or activity cosine similarity in the top 0.5 percentile. (a) Percent change in posts per day, (b) change in mean reposts per post, and (c) change in mean likes per post before versus after Musk purchased X. Changes are for not significant for coordinated accounts in (a) (Mann-Whitney U test p-values > 0.2) except for hashtag-based coordinated accounts (p-value = 0.04), but are significant for non-coordinated accounts (Mann-Whitney U test p-values <0.008). No changes are statistically significant for (b) or (c) (Mann-Whitney U test p-values ≥0.2) except for the change in reposts per post for hashtag-based coordinated accounts (Mann-Whitney U test p-value = 0.009). Error bars are standard errors. Because of the differences between how standard error and Mann-Whitney U test are calculated, there can be standard errors that overlap with zero and the results could still be statistically significant. (PDF) [file pone.0313293.s004.pdf]

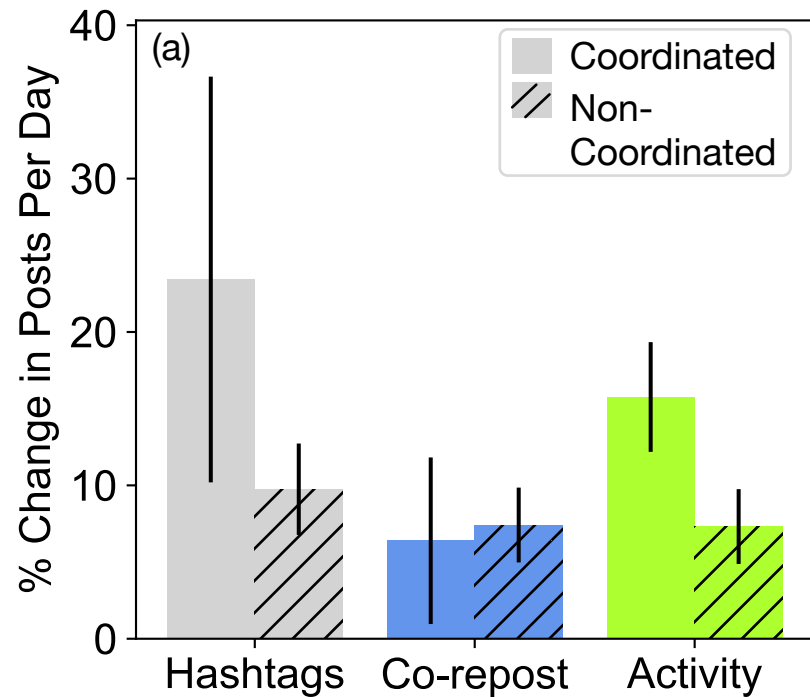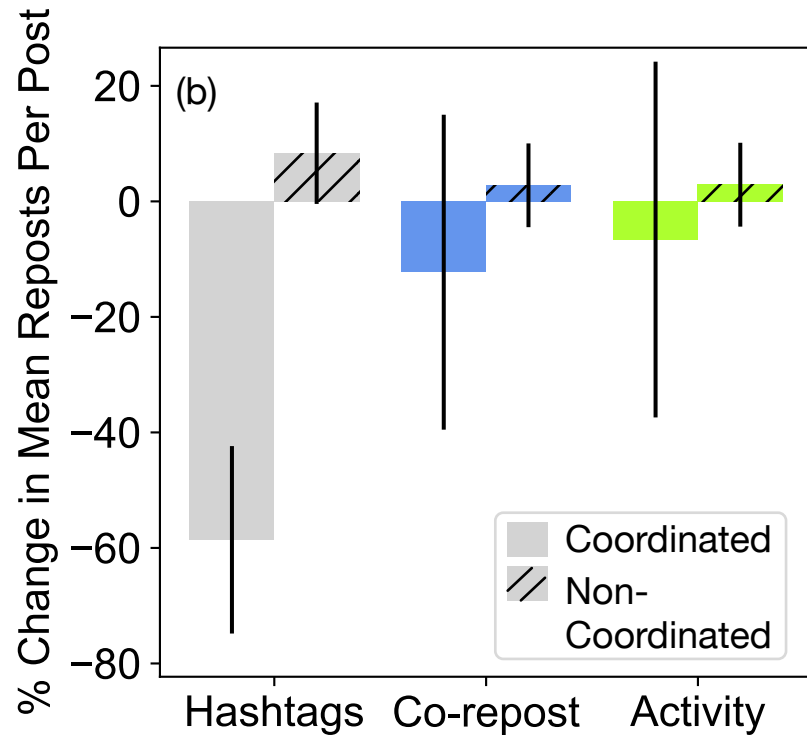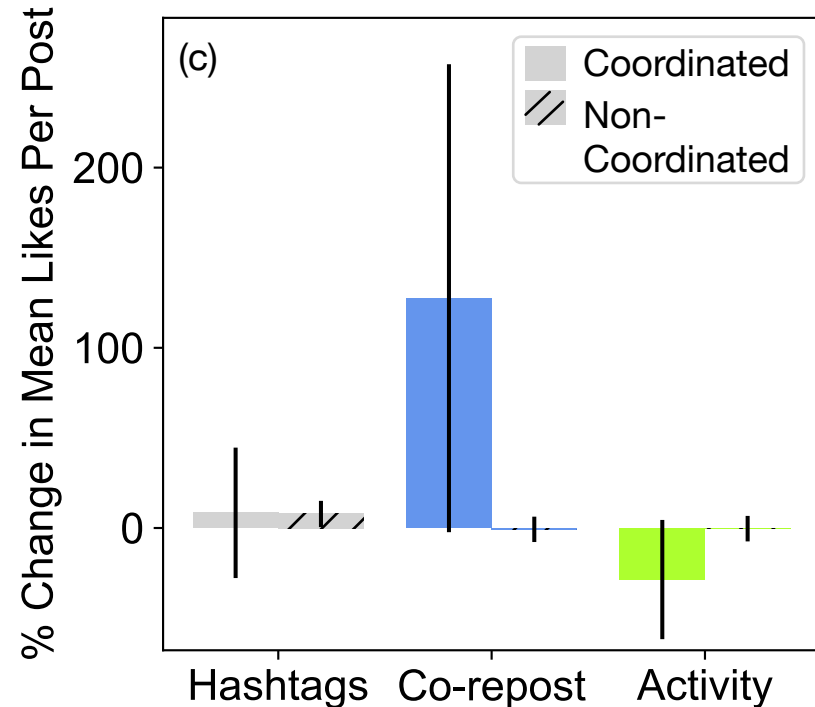

Supplement: S4 Fig — We use 3 different coordination metrics: hashtag sequence [32], co-repost [32, 33], and activity [32, 33]. Accounts are coordinated if they share a sequence of at least 7 hashtags in a post or have a co-retweet or activity cosine similarity in the top 0.01 percentile. (a) Percent change in posts per day, (b) change in mean reposts per post, and (c) change in mean likes per post before versus after Musk purchased X. Changes are for significant for all data (Mann-Whitney U test p-values <0.05) except for co-repost coordinated accounts (Mann-Whitney U test p-values = 0.38). No changes are statistically significant for (b) or (c) (Mann-Whitney U test p-values > 0.1) except for the change in reposts per post for hashtag based coordinated accounts (Mann-Whitney U test p-value = 0.002). Error bars are standard errors. Because of the differences between how standard error and Mann-Whitney U test are calculated, there can be standard errors that overlap with zero and the results could still be statistically significant. (PDF) [file pone.0313293.s005.pdf]

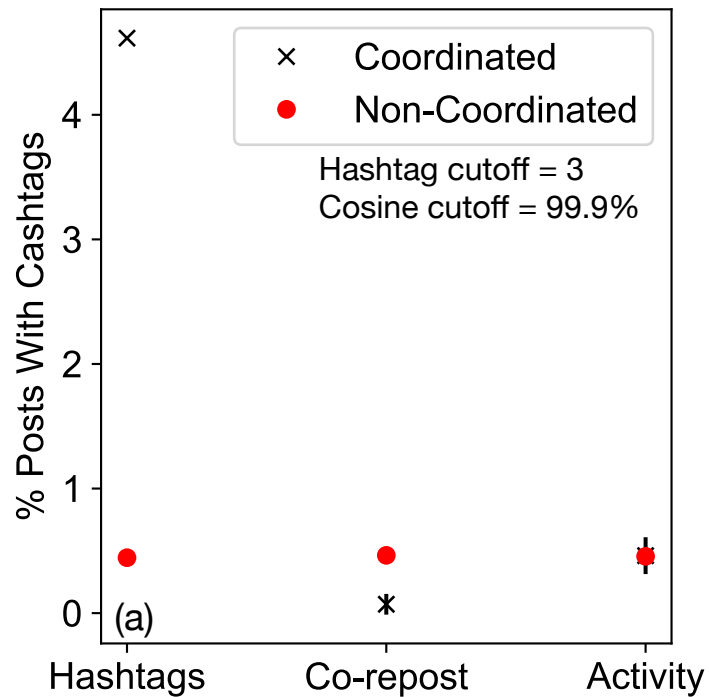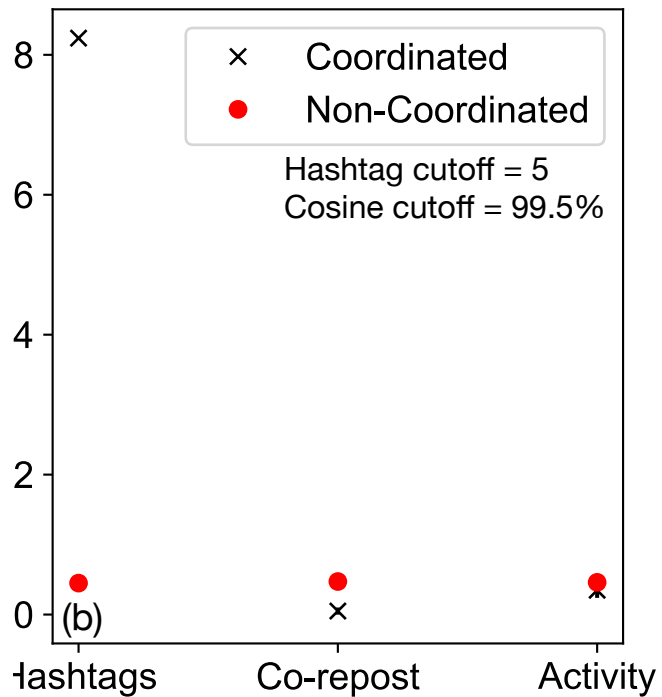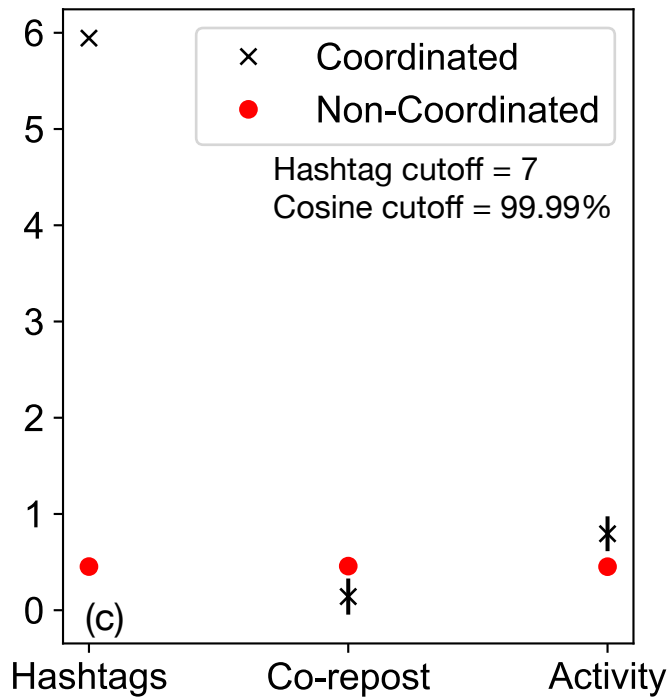

Supplement: S5 Fig — (a) 3-hashtag cutoff and cosine similarity percentage greater than 99.9%, (b) 5-hashtag cutoff and cosine similarity percentage greater than 99.5%, (c) 7-hashtag cutoff and cosine similarity percentage greater than 99.99%. We observe cashtags are significantly more common among hashtag similarity-based coordinated accounts. (PDF) [file pone.0313293.s006.pdf]
